# Supplementary material for: Induction of Male Sterility by Targeted Mutation of a Restorer-of-Fertility Gene with CRISPR/Cas9-Mediated Genome Editing in Brassica napus L
Source: Plants (Basel). 2022 Dec 13;11(24):3501. doi: 10.3390/plants11243501 (PMC9785856; doi:10.3390/plants11243501)
Supplement: Supplementary file 1 [file plants-11-03501-s001.zip › Supplementary Figures.pdf]

```
                           10         20         30         40         50         60         70         80         90        100
                   ....|....| ....|....| ....|....| ....|....| ....|....| ....|....| ....|....| ....|....| ....|....| ....|....|
BnaA09g45590D      TGAAATTCGT TCGGTGTGCG GATCTAGTTG TGGGTGAAAG GGCGAA---G AAGAATC--- ---ATCAAGT CA--CTGAAG ATGAAGATGG GAGTGGAGTT
At1g12620          TTTGTTTTAT TGTGTGTGAA AGAGGTGTAA TGAGCAGACA TTGGTGCTTG TGGAAGCCGA ACAAACGAAT CAGCACAAAA CCGAAGACGT GACTCCAATT
At1g12300          GCTGTTTTGT AGGGTTTGCA ATTTACACAC AGAGTGAGAG TGAGAGTTTG AAAAGG---- ---AACGATT CAGCTTGAGA TGGTGAAGTT GATGATACGG
Clustal Consensus       **  *    ** **             * *          *     *    *         * * * * **     *     *   *    **    *

                          110        120        130        140        150        160        170        180        190        200
                   ....|....| ....|....| ....|....| ....|....| ....|....| ....|....| ....|....| ....|....| ....|....| ....|....|
BnaA09g45590D      GTTCTTCTTC TTCTTCTAGG TTCTTTATTT TTCCAGATTT GAAACAAAAA CAGACAATGT TGTGTCGGAG ATTGGTACGT GAGCCTCGTA TCCCTCTGAG
At1g12620          TTTTTTTTTT TAAATCAGAG CTTTCAAATT CTTAAC--TT GAGTCATGCG GGGATTG-AT TCAGACACGT CTTCTCGAGA CAGGTACTCT AAGAACTGCT
At1g12300          AGATTGAGTT C---TCAAGT TTCGAAATTT GTTCAG--CC TCGTCTCCTG GAGACAG-GT ACTCTGAGAA TTGCTTTGAT TAATTGCCCA AA----TGAG
Clustal Consensus      *   *      **       *    * **  *  *          *          **     *             *          *    *           **

                          210        220        230        240        250        260        270        280        290        300
                   ....|....| ....|....| ....|....| ....|....| ....|....| ....|....| ....|....| ....|....| ....|....| ....|....|
BnaA09g45590D      TCTTGGAGGT ACTCTTACAG TTACAACTCA TCATTTGCTC TCTCGGAAGA TGTC-----T TGTTACAAAG AGAGATTGCG AAGTGGGATC GTCGATATTA
At1g12620          TTATTCTTGT CTTGCTATGG ACGAGTCTTT TCTAGTGTCA GTGATGGGAA AGGGAAAGTC TCCTATAGAG AGAGATTGAG AAGTGGGATT GTTGATATCA
At1g12300          CTATCTTT-- -TTGCTGCGA ACGCGGCTTC TCTGCTTTCA GCGATAGAAA TCTG------ TCTTATAGAG AGAGATTGAG AAGTGGGCTT GTCGATATTA
Clustal Consensus     *         *  *           **    **   *              *            *  ** * ** ******** * ******* *  ** ***** *

                          310        320        330        340        350        360        370        380        390        400
                   ....|....| ....|....| ....|....| ....|....| ....|....| ....|....| ....|....| ....|....| ....|....| ....|....|
BnaA09g45590D      AGAAAGATGA AGCTGTTGCT CTGTTTCAAA CTATGATTGT GTCTCGTCCT CTTCCT---- ---------- ---------- ---------- ----------
At1g12620          AGGAAGATGA TGCTGTTGAT CTGTTTCAAG AAATGACTAG GTCTCGTCCT CGTCCTCGTC TTATTGATTT CAGTAGATTG TTTAGTGTTG TTGCCAGAAC
At1g12300          AGGCAGATGA TGCTATTGAT CTGTTCCGAG ACATGATTCA CTCTCGTCCT CTTCCCACTG TTATAGATTT CAGTAGATTG TTTAGTGCCA TTGCCAAAAC
Clustal Consensus  **  ******  *** *** * ***** * *    **** *    *********  * ***

                          410        420        430        440        450        460        470        480        490        500
                   ....|....| ....|....| ....|....| ....|....| ....|....| ....|....| ....|....| ....|....| ....|....| ....|....|
BnaA09g45590D      ---------- ---------- ---------- -ACGCAAATG GAAATGAATG GGATTGCGCA TGACCTCTAC ACGCTGAGTA TTGTCATCAA CTGCTTCTGC
At1g12620          AAAGCAATAT GATCTAGTGT TAGATCTCTG CAAGCAAATG GAATTAAAGG GAATTGCGCA TAACCTCTAC ACTCTGAGTA TTATGATTAA TTGCTGTTGT
At1g12300          GAAACAGTAT GATCTTGTGT TAGCTCTCTG CAAGCAAATG GAATTAAAGG GAATCGCGCA TAACCTCTAC ACTCTGAGTA TTATGATTAA TTGCTTTTGT
Clustal Consensus                                   * ******* *** * ** * * ** ***** * ******** ** ******* ** * ** **  ****  **

                          510        520        530        540        550        560        570        580        590        600
                   ....|....| ....|....| ....|....| ....|....| ....|....| ....|....| ....|....| ....|....| ....|....| ....|....|
BnaA09g45590D      CGTCGCCGGA AACTC----- ---------- ---------- ---------- ---------- ---------- --GTCACTTT CTCAACTTTG ATTAACGGTT
At1g12620          CGATGTCGTA AACTCTCTTT GGCTTTTTCT GCTATGGGGA AGATCATTAA ACTTGGGTAT GAGCCTGATA CAGTCACATT TTCAACTTTG ATAAACGGAT
At1g12300          CGATGTCGTA AACTCTGTTT GGCTTTTTCT GCTATGGGGA AGATCATTAA ACTTGGGTAT GAGCCTAACA CTATCACATT TTCAACTTTA ATCAACGGAT
Clustal Consensus  **  * ** * *****                                                                **** **  ********  ** ***** *
```

```
                          610        620        630        640        650        660        670        680        690        700
                   ....|....| ....|....| ....|....| ....|....| ....|....| ....|....| ....|....| ....|....| ....|....| ....|....|
BnaA09g45590D      TATGTCTCAA GGGTCGAGTC TCTGAAGCTG TGGAGTTAGT TGATCGTATG GTGGAAATGA AGGTTATTCC AAATCTCATC ATACTTAACA CTCTTGTCAA
At1g12620          TATGTCTCGA GGGTAGAGTT TCCGAAGCTC TGGAATTAGT TGATCGAATG GTTGAAATGG GACATAAACC CACTCTCATA ACGCTTAACG CTCTGGTCAA
At1g12300          TATGTCTCGA GGGTAGAGTT TCCGAAGCTC TGGAATTAGT TGATCGAATG GTTGAAATGG GACATAAACC CGATCTCATA ACTATTAACA CTCTGGTCAA
Clustal Consensus  ******** * **** ****  ** ******  **** ***** ****** *** ** ******      **  **    ******  *   *****  **** *****

                          710        720        730        740        750        760        770        780        790        800
                   ....|....| ....|....| ....|....| ....|....| ....|....| ....|....| ....|....| ....|....| ....|....| ....|....|
BnaA09g45590D      TGGACTTTGT CTCCAAGATA GAGTGTCCGA AGCAGTGGCT TTGATAGATC GAATGGTGGC TAATGGATGT CAACCCGATC AGTTTACCTA TGGTCCGATT
At1g12620          TGGACTTTGT CTCAACGGTA AAGTCTCTGA TGCTGTGCTT TTGATTGATC GAATGGTTGA GACTGGCTTT CAACCCAATG AAGTAACTTA TGGACCGGTT
At1g12300          TGGACTTTGT CTCAGCGGTA AAGAGGCTGA AGCAATGCTT TTGATAGATA AAATGGTGGA ATATGGCTGT CAACCCAATG CAGTTACCTA CGGACCGGTT
Clustal Consensus  ********** ***   * **  **   * **  **  **  * ***** ***   ****** *     *** * * ****** **     * ** **  ** *** **

                          810        820        830        840        850        860        870        880        890        900
                   ....|....| ....|....| ....|....| ....|....| ....|....| ....|....| ....|....| ....|....| ....|....| ....|....|
BnaA09g45590D      TTGAACAGAA TGTGCAA--- ---------- ---------- ---------- -------ATG GAAGATCGAA AGGTCAAGCC --------TC AAGTA-----
At1g12620          TTAAAAGTAA TGTGTAAGTC CGGCCAAACT GCCCTTGCCA TGGAGTTGCT CAGAAAGATG GAAGAAAGAA AGATCAAGCT CGATGCAGTC AAATACAGTA
At1g12300          TTAAATGTAA TGTGTAAGTC CGGCCAAACT GCCCTTGCCA TGGAGTTGCT CAGAAAGATG GAAGAAAGAA ATATCAAGCT CGATGCAGTC AAATACAGTA
Clustal Consensus  ** **   ** **** **                                            *** *****  *** *  ******          ** ** **

                          910        920        930        940        950        960        970        980        990       1000
                   ....|....| ....|....| ....|....| ....|....| ....|....| ....|....| ....|....| ....|....| ....|....| ....|....|
BnaA09g45590D      ---------- ---------- ---------- ---------- ---------- ---------- ---------- ---------- ---------- ----------
At1g12620          TCATCATTGA TGGTCTGTGC AAAGACGGGA GCCTCGACAA TGCATTCAAC CTTTTCAATG AAATGGAAAT CAAAGGGTTC AAAGCTGATA TTATTATCTA
At1g12300          TCATCATTGA TGGTCTTTGC AAACACGGGA GCCTCGACAA TGCATTCAAC CTTTTCAATG AAATGGAAAT GAAAGGGATC ACAACAAATA TTATTACCTA
Clustal Consensus

                         1010       1020       1030       1040       1050       1060       1070       1080       1090       1100
                   ....|....| ....|....| ....|....| ....|....| ....|....| ....|....| ....|....| ....|....| ....|....| ....|....|
BnaA09g45590D      ---------- ---------- ---------- ---------- ---------- ---------- ---------- ---------- ---------- ----------
At1g12620          CACCACTCTC ATTAGAGGCT TTTGTTACGC TGGTAGATGG GATGATGGTG CCAAGTTGCT GAGGGATATG ATCAAAAGGA AAATCACACC AGACGTGGTC
At1g12300          CAACATTCTC ATTGGCGGCT TTTGTAATGC TGGTAGATGG GATGATGGTG CCAAGTTATT GAGGGATATG ATCAAGAGGA AAATCAACCC CAACGTTGTC
Clustal Consensus

                         1110       1120       1130       1140       1150       1160       1170       1180       1190       1200
                   ....|....| ....|....| ....|....| ....|....| ....|....| ....|....| ....|....| ....|....| ....|....| ....|....|
BnaA09g45590D      --------TG CGTTGATTGA TAGTTTTGTG AAAGAAGGAA AGCTTACTGA GGCTAAAGAG TTGTACAATG AGATGATCGT AAGAGGCTTA GATCCTAATA
At1g12620          GCTTTCAGCG CGTTAATAGA TTGTTTTGTG AAAGAGGGAA AGCTTCGAGA GGCTGAAGAA CTGCACAAGG AGATGATACA ACGAGGCATA TCTCCTGATA
At1g12300          ACATTCAGTG TTTTGATTGA TAGTTTTGTG AAAGAGGGAA AGCTTCGAGA GGCCGAAGAA CTGCACAAGG AGATGATACA TCGAGGCATA GCTCCTGATA
Clustal Consensus           *   ** ** ** * ******** ***** **** *****   ** ***  ****   ** **** * *******      ***** **   **** ***
```

```
                            1210       1220       1230       1240       1250       1260       1270       1280       1290       1300
                    ....|....| ....|....| ....|....| ....|....| ....|....| ....|....| ....|....| ....|....| ....|....| ....|....|
BnaA09g45590D       CCATTACATA TGGTTCTCTG ATACATGGGC TGTGCATGGA GAATCGCTTA GATGAGGCCA ACCAGATGTT GGATCTGATG GTTAGCAAGG GATGCGATCC
At1g12620           CTGTTACGTA CACTTCTTTG ATAGATGGGT TTTGCAAGGA GAACCAGCTA GATAAGGCCA ACCACATGCT GGATCTGATG GTTAGCAAAG GATGCGGTCC
At1g12300           CTATCACATA CACTTCTTTG ATAGATGGGT TTTGCAAGGA GAACCACCTA GATAAGGCCA ACCAGATGGT GGATCTGATG GTTAGCAAAG GATGCGATCC
Clustal Consensus   *  * ** **    **** ** *** *****  * **** *** *** *   ** *** ****** **** *** * ********** ******** * ****** ***

                            1310       1320       1330       1340       1350       1360       1370       1380       1390       1400
                    ....|....| ....|....| ....|....| ....|....| ....|....| ....|....| ....|....| ....|....| ....|....| ....|....|
BnaA09g45590D       TGATATTTGG ACGTATAATA TCCTTATAAA CGGGTATTGT AAGGCTAAAC TGGTTGATGA AGGTATGAGA CTTTTCCGCA AAATGTCTCT GAGAGGAGTG
At1g12620           TAATATCAGG ACGTTTAATA TCCTCATAAA TGGATATTGT AAGGCTAATC TGATTGACGA TGGTTTGGAA CTCTTCCGCA AAATGTCTTT GAGAGGAGTG
At1g12300           TAATATCAGG ACGTTTAATA TCCTCATAAA TGGATATTGT AAGGCTAATC GAATTGACGA TGGTTTGGAA CTCTTCCGCA AAATGTCTTT GAGAGGAGTG
Clustal Consensus   * ****  ** **** ***** **** *****  ** ****** ******** *    **** **  *** **  * ** ******* ******** * **********

                            1410       1420       1430       1440       1450       1460       1470       1480       1490       1500
                    ....|....| ....|....| ....|....| ....|....| ....|....| ....|....| ....|....| ....|....| ....|....| ....|....|
BnaA09g45590D       GTTGCAGATA CAGTCACTTA TAGCAGTCTC ATTCAAGGGT TTTGTCAATC AGGAAAACTT AAAGTCGCCA AAGAACTCTT CCAAGAGATG GTCTCTCAAG
At1g12620           GTTGCAGATA CGGTTACTTA TAACACTCTT ATCCAAGGAT TTTGTGAATT GGGAAAACTT GAAGTTGCCA AAGAACTCTT CCAAGAGATG GTTTCTCGTC
At1g12300           GTTGCAGATA CAGTTACTTA TAACACTCTT ATCCAAGGAT TTTGTGAATT GGGAAAACTT AATGTTGCCA AAGAACTCTT CCAAGAGATG GTTTCTCGTA
Clustal Consensus   ********** * ** ***** ** ** ***  ** ***** * ***** ***   *********  * ** **** ********** ********** ** ****

                            1510       1520       1530       1540       1550       1560       1570       1580       1590       1600
                    ....|....| ....|....| ....|....| ....|....| ....|....| ....|....| ....|....| ....|....| ....|....| ....|....|
BnaA09g45590D       GTGTTCATCC TAATATTGTA ACCTACAAAA TTTTGATGGA TGGA------ ---------- ---------- ---------- ---------- ----------
At1g12620           GTGTTCGTCC TGATATTGTG TCCTACAAAA TTTTGCTCGA TGGGTTGTGT GACAATGGGG AACCAGAAAA AGCGCTGGAA ATATTTGAAA AAATAGAGAA
At1g12300           AAGTTCCTCC TAATATTGTG ACCTACAAAA TTTTGCTGGA TGGGTTGTGT GACAATGGGG AATCAGAAAA AGCGTTGGAA ATATTTGAAA AAATAGAGAA
Clustal Consensus     **** *** * *******   ********* ***** * ** ***

                            1610       1620       1630       1640       1650       1660       1670       1680       1690       1700
                    ....|....| ....|....| ....|....| ....|....| ....|....| ....|....| ....|....| ....|....| ....|....| ....|....|
BnaA09g45590D       ---------- ---------- ---------- ---------- ---------- ---------- -------GTC GATGAAGCTT GGGATCTATT TTGTAGCCTC
At1g12620           GAGTAAGATG GAGCTTGATA TTGGTATCTA TAACATCATC ATTCATGGGA TGTGCAATGC TAGTAAGGTG GATGATGCTT GGGATTTATT CTGTAGCCTC
At1g12300           GAGTAAGATG GAGCTTGATA TTGGTATCTA TAACATCATC ATTCACGGGA TGTGCAATGC TAGTAAGGTG GATGATGCTT GGGATTTATT CTGTAGCCTC
Clustal Consensus                                                                              **  ***** **** ***** ****  *********

                            1710       1720       1730       1740       1750       1760       1770       1780       1790       1800
                    ....|....| ....|....| ....|....| ....|....| ....|....| ....|....| ....|....| ....|....| ....|....| ....|....|
BnaA09g45590D       CCTCTCAAAG AAGTGAAGCC TGATGTCAAA ACATACACCA TAATGATTGG AGGACTATGT AAGAAAGGCT CGATGTCTAA AGCGGGCCTG TTATTTAAGA
At1g12620           CCTCTCAAAG GAGTGAAGCC CGATGTCAAA ACATACAATA TAATGATTGG GGGACTGTGT AAGAAAGGCT CACTGTCTGA AGCAGACCTG TTGTTTAGAA
At1g12300           CCCCTCAAAG GAGTGAAGCC CGGTGTCAAA ACATACAATA TAATGATTGG GGGACTGTGT AAGAAAGGCC CATTGTCTGA AGCGGAACTG TTGTTTAGAA
Clustal Consensus   ** *******  *********  * ******* *******  * **********  ***** *** *********  *  ***** * *** *  *** ** ****  *
```

```
                              1810       1820       1830       1840       1850       1860       1870       1880       1890       1900
                      ....|....| ....|....| ....|....| ....|....| ....|....| ....|....| ....|....| ....|....| ....|....| ....|....|
BnaA09g45590D         AAATGGGAGA GGATGGGATT GCACCAGATG ATTGTACATG GAACACACTA ATCCGAGCTC ATCTCCGAGG TGGTGACTTA GCAACATCAG CTAAACTTAT
At1g12620             AAATGGAAGA GGATGGGCAT TCGCCAAATG GTTGTACATA CAACATACTC ATCAGGGCAC ATCTTGGAGA AGGTGATGCA ACCAAATCAG CTAAACTTAT
At1g12300             AAATGGAAGA GGATGGGCAT GCGCCAGATG GTTGGACATA CAACATACTC ATCAGGGCAC ATCTTGGAGA TGGTGATGCA ACCAAATCAG TTAAACTTAT
Clustal Consensus     ****** *** *******  *  * *** ***  *** ****   **** ***  *** * ** * ****  ***    *****   *  * * *****  *********

                              1910       1920       1930       1940       1950       1960       1970       1980       1990       2000
                      ....|....| ....|....| ....|....| ....|....| ....|....| ....|....| ....|....| ....|....| ....|....| ....|....|
BnaA09g45590D         AGAAGAAATG AAGAGTTGTG GGTTCTCTGC AGATGCTTCC ACGATAAAGA TTGTTATGGA TATGTTATCG GATGGTAGAA TGAAGAAAAG CTTTCTGGAT
At1g12620             TGAAGAAATA AAGAGGTGCG GGTTCTCAGT TGATGCTTCC ACGGTAAAGA TGGTTGTCGA TATGTTATCG GATGGTAGAT TGAAGAAAAG CTTTTTGGAT
At1g12300             TGAAGAACTG AAGAGGTGCG GGTTCTCAGT TGATGCTTCC ACTATAAAGA TGGTTATCGA TATGTTATCG GATGGTAGAT TGAAGAAAAG CTTTTTGGAT
Clustal Consensus      ****** *  ***** ** * ******* *   ********* **  ****** * *** * ** ********** *********  ********** **** *****

                              2010       2020       2030       2040       2050       2060       2070       2080       2090       2100
                      ....|....| ....|....| ....|....| ....|....| ....|....| ....|....| ....|....| ....|....| ....|....| ....|....|
BnaA09g45590D         TTGCTTTCTT AG-------- ---------- ---------- ---------- ---------- ---------- ---------- ---------- ----------
At1g12620             ATGCTTTCTT AGAATTTTGG TGTTTACATC ATATTACCGT CTCAGAGCAT TGAAAGACTA AGTTGGAGTT TCATATGTGA CAACATTGAT GTACACAACA
At1g12300             ATGCTTTCTT AGAATGTTTG ATCTTA-ATC A-ACCAGAGA TAAAGCTAAC TATTTCTCTA AGTATCACAA CTGTA-GCAA ATCTACTAGC TTTTGGAACC
Clustal Consensus      ********* **

                              2110       2120       2130       2140       2150       2160       2170       2180       2190       2200
                      ....|....| ....|....| ....|....| ....|....| ....|....| ....|....| ....|....| ....|....| ....|....| ....|....|
BnaA09g45590D         ---------- ---------- ---------- ---------- ---------- ---------- ---------- ---------- ---------- ----------
At1g12620             TTTTTCTTAA GCAGATCGAC CACCAAGATT TATAACCATT CTCCGGGC-A TGAAAGCAAG CTCATAATTT CCTTATCACT CTGTTC--AT CTGTCTACAT
At1g12300             AACCTGAAAA ACCTTCAGAC TCTTGGAACT GGAAGTGTCT CTTAAGGTTG CGTTGTTTAG CAGAAAACTT TGTCAGGTGT AAGATTGGAG ATGGAAAGAC
Clustal Consensus

                              2210       2220       2230       2240       2250       2260       2270       2280       2290       2300
                      ....|....| ....|....| ....|....| ....|....| ....|....| ....|....| ....|....| ....|....| ....|....| ....|....|
BnaA09g45590D         ---------- ---------- ---------- ---------- ---------- ---------- ---------- ---------- ---------- ----------
At1g12620             TGCT--TTTC AGATTGTACT TGCTAAATTC TG-------- CACAAATATG TATAG-GTAA ATGATATAAT TTGTAGCTTA AACACACCAT CGCTATCAAA
At1g12300             TGCTAGTTTC TGGTT-TGAT AACTGGACTC CTTTTGGTCC CCTAATCAAG TTCATTGGAA CAGATGGCCC CTGTAGTTGA GAGCCCCTTT GAATGCTAAG
Clustal Consensus
```

Figure S1. Sequence alignment of *BnaRFL11* with *Arabidopsis* homologs genes. Gene sequence alignment from *B. napus* (*BnaA09g45590D*) and *A. thaliana* (*AT1G12300, AT1G12620*). The base differences are highlighted in grey boxes. Star show the similarities between each copy and portion without star show polymorphism.

```
                                10         20         30         40         50         60         70         80         90        100
```

```
                    ....|....| ....|....| ....|....| ....|....| ....|....| ....|....| ....|....| ....|....| ....|....| ....|....|
BnaA09g45590D       YKERLRSGIV DIKKDEAVAL FQTMIVSRPL PTQMEMNGIA HDLYTLSIVI NCFCRRRKL- ---------- --------VT FSTLINGLCL KGRVSEAVEL
AT1G12300           YRERLRSGLV DIKADDAIDL FRDMIHSRPL PKQMELKGIA HNLYTLSIMI NCFCRCRKLC LAFSAMGKII KLGYEPNTIT FSTLINGLCL EGRVSEALEL
AT1G12620           YRERLRSGIV DIKEDDAVDL FQEMTRSRPR PKQMELKGIA HNLYTLSIMI NCCCRCRKLS LAFSAMGKII KLGYEPDTVT FSTLINGLCL EGRVSEALEL
Clustal Consensus   *:******:* *** *:*: * *: *  ***  *.***::*** *:******:* ** ** ***                     :* ********** :******:**

                           110        120        130        140        150        160        170        180        190        200
                    ....|....| ....|....| ....|....| ....|....| ....|....| ....|....| ....|....| ....|....| ....|....| ....|....|
BnaA09g45590D       VDRMVEMKVI PNLIILNTLV NGLCLQDRVS EAVALIDRMV ANGCQPDQFT YGPILNRMCK WKIERSSLKY ALIDSFVKEG KLTEAKELYN EMIVRGLDPN
AT1G12300           VDRMVEMGHK PDLITINTLV NGLCLSGKEA EAMLLIDKMV EYGCQPNAVT YGPVLNVMCK RNIKLDAVKY VLIDSFVKEG KLREAEELHK EMIHRGIAPD
AT1G12620           VDRMVEMGHK PTLITLNALV NGLCLNGKVS DAVLLIDRMV ETGFQPNEVT YGPVLKVMCK RKIKLDAVKY ALIDCFVKEG KLREAEELHK EMIQRGISPD
Clustal Consensus   *******    * ** :*:** *****..: : :*: ***:**   * **: .* ***:*: ***  :*: .::** .***.***** ** **:**:: *** **: *:

                           210        220        230        240        250        260        270        280        290        300
                    ....|....| ....|....| ....|....| ....|....| ....|....| ....|....| ....|....| ....|....| ....|....| ....|....|
BnaA09g45590D       TITYGSLIHG LCMENRLDEA NQMLDLMVSK GCDPDIWTYN ILINGYCKAK LVDEGMRLFR KMSLRGVVAD TVTYSSLIQG FCQSGKLKVA KELFQEMVSQ
AT1G12300           TITYTSLIDG FCKENHLDKA NQMVDLMVSK GCDPNIRTFN ILINGYCKAN RIDDGLELFR KMSLRGVVAD TVTYNTLIQG FCELGKLNVA KELFQEMVSR
AT1G12620           TVTYTSLIDG FCKENQLDKA NHMLDLMVSK GCGPNIRTFN ILINGYCKAN LIDDGLELFR KMSLRGVVAD TVTYNTLIQG FCELGKLEVA KELFQEMVSR
Clustal Consensus   *:** ***.* :* **:**:* *:*:****** **.*:* *:* *********:  :*:*:.*** ********** ****.:**** **: ***:** *********:

                           310        320        330        340        350        360        370        380        390        400
                    ....|....| ....|....| ....|....| ....|....| ....|....| ....|....| ....|....| ....|....| ....|....| ....|....|
BnaA09g45590D       GVHPNIVTYK ILMDG--VDE AWDLFCSLPL KEVKPDVKTY TIMIGGLCKK GSMSKAGLLF KKMGEDGIAP DDCTWNTLIR AHLRGGDLAT SAKLIEEMKS
AT1G12300           KVPPNIVTYK ILLDGLKVDD AWDLFCSLPL KGVKPGVKTY NIMIGGLCKK GPLSEAELLF RKMEEDGHAP DGWTYNILIR AHLGDGDATK SVKLIEELKR
AT1G12620           RVRPDIVSYK ILLDGLKVDD AWDLFCSLPL KGVKPDVKTY NIMIGGLCKK GSLSEADLLF RKMEEDGHSP NGCTYNILIR AHLGEGDATK SAKLIEEIKR
Clustal Consensus    * *:**:** **:**  **: ********** * ***.**** .********* *.:*:* *** :** *** :* :. *:* *** ***  ** :. *.*****:*

                           410        420        430
                    ....|....| ....|....| ....|....|.
BnaA09g45590D       CGFSADASTI KIVMDMLSDG RMKKSFLDLL S
AT1G12300           CGFSVDASTI KMVIDMLSDG RLKKSFLDML S
AT1G12620           CGFSVDASTV KMVVDMLSDG RLKKSFLDML S
Clustal Consensus   ****.****: *:*:****** *:******:* *
```

Figure S2. Protein sequence alignment from *B. napus* (*BnaA09g45590D*) and *A. thaliana* (*AT1G12300, AT1G12620*). The base differences are highlighted in grey boxes. Star show the similarities between each copy and portion without star show polymorphism.

***BnaRFL11* targets**

```
                        10         20         30         40         50         60         70         80         90        100
                ....|....| ....|....| ....|....| ....|....| ....|....| ....|....| ....|....| ....|....| ....|....| ....|....|
BnaA09g45590D   AAATCATCGA TGAAATTCGT TCGGTGTGCG GATCTAGTTG TGGGTGAAAG GGCGAAGAAG AATCATCAAG TCACTGAAGA TGAAGATGGG AGTGGAGTTG

                       110        120        130        140        150        160        170        180     S1  190        200
                ....|....| ....|....| ....|....| ....|....| ....|....| ....|....| ....|....| ....|....| ....|....| ....|....|
BnaA09g45590D   TTCTTCTTCT TCTTCTAGGT TCTTTATTTT TCCAGATTTG AAACAAAAAC AGACAATGTT GTGTCGGAGA TTGGTACGTG AGCCTCGTAT CCCTCTGAGT

                       210        220        230        240        250        260        270        280        290        300
                ....|....| ....|....| ....|....| ....|....| ....|....| ....|....| ....|....| ....|....| ....|....| ....|....|
BnaA09g45590D   CTTGGAGGTA CTCTTACAGT TACAACTCAT CATTTGCTCT CTCGGAAGAT GTCTTGTTAC AAAGAGAGAT TGCGAAGTGG GATCGTCGAT ATTAAGAAAG

                       310        320        330        340        350        360        370        380        390        400
                ....|....| ....|....| ....|....| ....|....| ....|....| ....|....| ....|....| ....|....| ....|....| ....|....|
BnaA09g45590D   ATGAAGCTGT TGCTCTGTTT CAAACTATGA TTGTGTCTCG TCCTCTTCCT ACGCAAATGG AAATGAATGG GATTGCGCAT GACCTCTACA CGCTGAGTAT

                       410        420        430        440        450        460        470        480        490        500
                ....|....| ....|....| ....|....| ....|....| ....|....| ....|....| ....|....| ....|....| ....|....| ....|....|
BnaA09g45590D   TGTCATCAAC TGCTTCTGCC GTCGCCGGAA ACTCGTCACT TTCTCAACTT TGATTAACGG TTTATGTCTC AAGGGTCGAG TCTCTGAAGC TGTGGAGTTA

                       510        520        530        540        550        560        570        580        590        600
                ....|....| ....|....| ....|....| ....|....| ....|....| ....|....| ....|....| ....|....| ....|....| ....|....|
BnaA09g45590D   GTTGATCGTA TGGTGGAAAT GAAGGTTATT CCAAATCTCA TCATACTTAA CACTCTTGTC AATGGACTTT GTCTCCAAGA TAGAGTGTCC GAAGCAGTGG

                       610        620        630        640        650        660        670        680     S1  690        700
                ....|....| ....|....| ....|....| ....|....| ....|....| ....|....| ....|....| ....|....| ....|....| ....|....|
BnaA09g45590D   CTTTGATAGA TCGAATGGTG GCTAATGGAT GTCAACCCGA TCAGTTTACC TATGGTCCGA TTTTGAACAG AATGTGCAAA TGGAAGATCG AAAGGTCAAG

                       710        720        730        740        750        760        770        780        790        800
                ....|....| ....|....| ....|....| ....|....| ....|....| ....|....| ....|....| ....|....| ....|....| ....|....|
BnaA09g45590D   CCTCAAGTAT GCGTTGATTG ATAGTTTTGT GAAAGAAGGA AAGCTTACTG AGGCTAAAGA GTTGTACAAT GAGATGATCG TAAGAGGCTT AGATCCTAAT

                       810        820        830        840        850        860        870        880        890        900
                ....|....| ....|....| ....|....| ....|....| ....|....| ....|....| ....|....| ....|....| ....|....| ....|....|
BnaA09g45590D   ACCATTACAT ATGGTTCTCT GATACATGGG CTGTGCATGG AGAATCGCTT AGATGAGGCC AACCAGATGT TGGATCTGAT GGTTAGCAAG GGATGCGATC

                       910        920        930        940        950        960        970        980        990       1000
                ....|....| ....|....| ....|....| ....|....| ....|....| ....|....| ....|....| ....|....| ....|....| ....|....|
BnaA09g45590D   CTGATATTTG GACGTATAAT ATCCTTATAA ACGGGTATTG TAAGGCTAAA CTGGTTGATG AAGGTATGAG ACTTTTCCGC AAAATGTCTC TGAGAGGAGT
```

```
                            1010      1020      1030      1040      1050      1060      1070      1080      1090      1100
                    ....|....| ....|....| ....|....| ....|....| ....|....| ....|....| ....|....| ....|....| ....|....| ....|....|
BnaA09g45590D       GGTTGCAGAT ACAGTCACTT ATAGCAGTCT CATTCAAGGG TTTTGTCAAT CAGGAAAACT TAAAGTCGCC AAAGAACTCT TCCAAGAGAT GGTCTCTCAA

                            1110      1120      1130      1140      1150      1160      1170      1180      1190      1200
                    ....|....| ....|....| ....|....| ....|....| ....|....| ....|....| ....|....| ....|....| ....|....| ....|....|
BnaA09g45590D       GGTGTTCATC CTAATATTGT AACCTACAAA ATTTTGATGG ATGGAGTCGA TGAAGCTTGG GATCTATTTT GTAGCCTCCC TCTCAAAGAA GTGAAGCCTG

                            1210      1220      1230      1240      1250      1260      1270      1280      1290      1300
                    ....|....| ....|....| ....|....| ....|....| ....|....| ....|....| ....|....| ....|....| ....|....| ....|....|
BnaA09g45590D       ATGTCAAAAC ATACACCATA ATGATTGGAG GACTATGTAA GAAAGGCTCG ATGTCTAAAG CGGGCCTGTT ATTTAAGAAA ATGGGAGAGG ATGGGATTGC
                            1310      1320      1330      1340      1350      1360      1370      1380      1390      1400
                    ....|....| ....|....| ....|....| ....|....| ....|....| ....|....| ....|....| ....|....| ....|....| ....|....|
BnaA09g45590D       ACCAGATGAT TGTACATGGA ACACACTAAT CCGAGCTCAT CTCCGAGGTG GTGACTTAGC AACATCAGCT AAACTTATAG AAGAAATGAA GAGTTGTGGG

                            1410      1420      1430      1440      1450      1460      1470      1480      1490
                    ....|....| ....|....| ....|....| ....|....| ....|....| ....|....| ....|....| ....|....| ....|....|
BnaA09g45590D       TTCTCTGCAG ATGCTTCCAC GATAAAGATT GTTATGGATA TGTTATCGGA TGGTAGAATG AAGAAAAGCT TTCTGGATTT GCTTTCTTAG
```

Figure S3. Sequence alignment of single functional *RFL11* gene copy in Westar. The target sequences are shown in underline with the PAM highlighted in red.

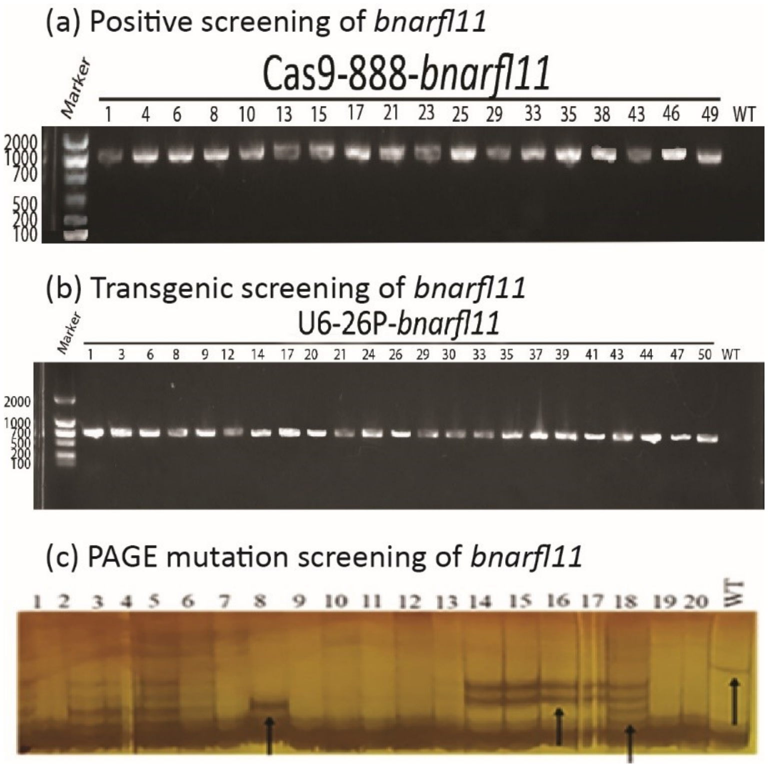

Figure. S4 Positive, Transgenic and mutation screening in *bnarfl11*. (a) Cas9 specific primers used for positive screening of *bnarfl11*. (**b)** Universal primers used for transgenic screening of *bnarfl11*. (**c)** Polyacrylamide gel electrophoresis for mutation detection, numerous plants showed various bands that differ from the band on non-denaturing PAGE gels in wild-type plants (WT), displaying target sites with some mutation.

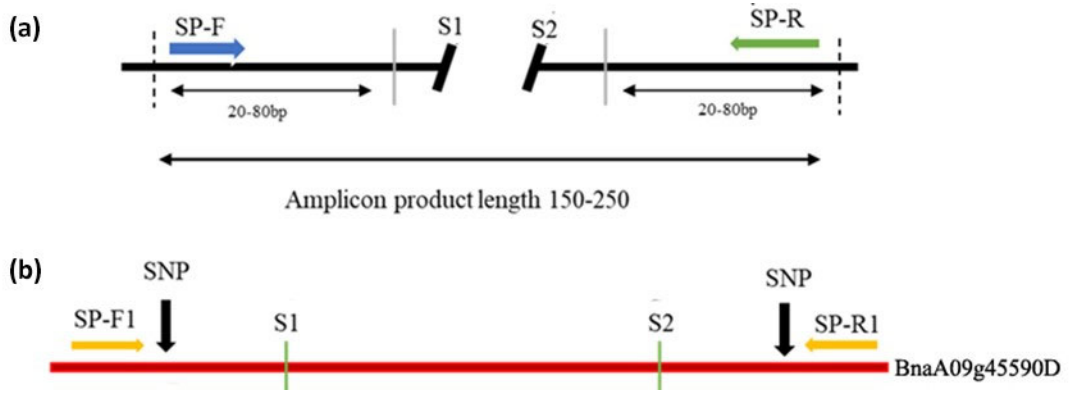

Figure S5 The *BnaRFL11* gene contains a single copy with two target sites S1 and S2. (a) in a single copy, forward and reverse primers covered single target sites, and both primers were designed within 20-80 nucleotides from the target sites. The length of the specific primer pair (SP F/R) was 150-250 bp. **(b)** Site-specific primers were designed for each target based on SNP differences.

10 20 30 40 50 60 70 80 90 100

```
                   ....|....|....|....|....|....|....|....|....|....|....|....|....|....|....|....|....|....|....|....|
RFL11              TGATAAATTCGTGGTGTGGATCTAGTTGTGGGTGAAAGGGAAGAAATCATCAAGTCATGAAGATGGGAGTGGAGTTGTTCTTCTTCTTCTGGTTTATTTA 100
RFL6               TGATAAATTCGTGGTGTGGATGTTTTTGTGG-TGAAAGGGAAGAAATCA--AAGTCATGAAGACGGGAGTGGAGTTGTTCTTCTTCTTCTGGTTTATTTA 97
ClustalConsensus   ********************* *  ****** *****************  ************ ************************************ 93

                            110       120       130       140       150       160       170       180       190       200
                   ....|....|....|....|....|....|....|....|....|....|....|....|....|....|....|....|....|....|....|....|
RFL11              GATTTGAAAAAAACAGACAATGTTGTGTCGGAGATTGGTACGTGAGCCTCGTATCCCTCTGAGTCTTGGAGGTACTCTTACAGTTACATGTCTTGT--TA 198
RFL6               GATTTGAAAAAAACAAACAATGTTGTGTCGGAGATTGGTGCTTGTGTCTCGTATCCCCCTGAGTCCTGTTGGTACTCTTGCAACTCCATCTCTCATCTTA 197
ClustalConsensus   *************** *********************** * ** * ********** ******* **  ********* **  * *** ***  *  ** 175

                            210       220       230       240       250       260       270       280       290       300
                   ....|....|....|....|....|....|....|....|....|....|....|....|....|....|....|....|....|....|....|....|
RFL11              CAAAGAGAGATTGCGAAGTGGGATCGTCGATATTAAGAAAGATGAAGCTGTTGCTCTGTTTCAAACTATGATTGTGTCTCGTCCTCTTCCTACG---CAA 295
RFL6               CAAAGAGAGACTGAGAAGTGGTCTGGTCGATATCAAGAAGAAGGATGCTGTAGCTCTGTTTCAGTCCATGATTAGGTCTCGTCCTCTTCCTACGAAGCAA 297
ClustalConsensus   ********** ** *******  * ******** *****  * ** ***** ***********  * ******  ******************   *** 256

                            310       320       330       340       350       360       370       380       390       400
                   ....|....|....|....|....|....|....|....|....|....|....|....|....|....|....|....|....|....|....|....|
RFL11              ATGGAAATGAATGGGATTGCGCATGACCTCTACACGCTGAGTATTGTCATCAACTGCTTCTGCCGTCGCCGGAAACTCGTCACTTTCTCAACTTTGATTA 395
RFL6               ATGGAACTGCAAGGGATTGCACATAGCATTTACACGCTGAGTATTATGATCAATTGCTTCTGCCGCCTCCAGAAACTCGTCACATTCTCAACTTTGATCA 397
ClustalConsensus   ****** ** * ******** ***  * * *************** * ***** *********** * ** ************ ************* * 340

                            410       420       430       440       450       460       470       480       490       500
                   ....|....|....|....|....|....|....|....|....|....|....|....|....|....|....|....|....|....|....|....|
RFL11              ACGGTTTATGTCTGGGTCGAGTCTCTGAAGCTGTGGAGTTAGTTGATCGTATGGTGGAAATGAAGGTTATTCCAAATCTCATCATACTTAACACTCTTGT 495
RFL6               ACGGTTTATGTCTGGGTAGAGTTTCCAAAGCTGTGGAGTTAGTTGATCATATGGTAGATATGAAGGTTATTCCAAATCTCATCATACTTAACACTATTGT 497
ClustalConsensus   ***************** **** **  ********************* ****** ** ************************************ **** 432

                            510       520       530       540       550       560       570       580       590       600
                   ....|....|....|....|....|....|....|....|....|....|....|....|....|....|....|....|....|....|....|....|
RFL11              CAATGGACTTTGTCTCCAAGATAGAGTGTCCGAAGCAGTGGCTTTGATAGATCGAATGGTGGCTAATGGATGTCAACCCGATTACCTATGGTCCGATTTT 595
RFL6               CAATGGGCTTTGTCTCCAAGATAGACTCTCTGAAGCAATGTCTTTGATACATCGAATGTTGGCTAATGGGTGCCAACCCGATTACATATGGTCCGGTTTT 597
ClustalConsensus   ****** ****************** * ** ****** ** ******** ******** ********** ** ************ ********* **** 520

                            610       620       630       640       650       660       670       680       690       700
                   ....|....|....|....|....|....|....|....|....|....|....|....|....|....|....|....|....|....|....|....|
RFL11              GAACAGAATGTGCAAATGGAAGATCGAAAGGTCAAGCCTCAAGTATGCGTTGATTGATAGTTTTGTGAAAGAAGGAAAGCTTACTGAGGCTAAAGAGTTG 695
RFL6               GAACAGAATGTGTAAATGGAACTTAGAAAGATCAAGCCTCAAGTATGCTTTGATTGATAGTTTTGTGAAAGAAGGACAGCTTACCGAGGCTAAAAAAATTG 697
ClustalConsensus   ************ ********  * ***** ***************** *************************** ******* ********* * *** 610

                            710       720       730       740       750       760       770       780       790       800
                   ....|....|....|....|....|....|....|....|....|....|....|....|....|....|....|....|....|....|....|....|
RFL11              TACAATGAGATGATCGTAAGAGGCTTAGATCCTAATACCATTACATATGGTTCTCTGATACATGGGCTGTGCATGGAGAATCGCTTAGATGAGGCCAACC 795
RFL6               TACAATGAGATGATCACAAGAGGCACAGATCCAAATATCATTACATATAACTCTTTGATATATGGGATGTGCATGGACAACTGCCTAGATGAGGCCAACC 797
ClustalConsensus   ***************  *******  ****** **** **********   *** ***** ***** ********** **  ** *************** 694

                            810       820       830       840       850       860       870       880       890       900
                   ....|....|....|....|....|....|....|....|....|....|....|....|....|....|....|....|....|....|....|....|
RFL11              AGATGTTGGATCTGATGGTTAGCAAGGGATGCGATCCTGATATTTGGACGTATAATATCCTTATAAACGGGTATTGTAAGGCTAAACTGGTTGATGAAGG 895
RFL6               AGATGCTGGATATGATGGTTAGCAAGGGATTCTATCCTGATATTGTGACGTTTAATATCCTTATCAACGGATACTGTAAGGCTAAACAGGTCGATGAAGG 897
```

```
ClustalConsensus  ***** ***** ****************** * ***********  ***** ************ ***** ** ************* *** ******** 782

                         910       920       930       940       950       960       970       980       990      1000
                  ....|....|....|....|....|....|....|....|....|....|....|....|....|....|....|....|....|....|....|....|
RFL11             TATGAGACTTTTCCGCAAAATGTCTCTGAGAGGAGTGGTTGCAGATACAGTCACTTATAGCAGTCTCATTCAAGGGTTTTGTCAATCAGGAAAACTTAAA 995
RFL6              TACGAGACTTTTCCGCAATATGTGTTTAAGAGGAGTGGTTGCTGATACAGTCACTTATAACACTCTCATCCAAGGGTTTTGTCAATCGGGAAAACTTAAT 997
ClustalConsensus  ** *************** **** * * ************** **************** ** ****** ***************** ***********  871

                        1010      1020      1030      1040      1050      1060      1070      1080      1090      1100
                  ....|....|....|....|....|....|....|....|....|....|....|....|....|....|....|....|....|....|....|....|
RFL11             GTCGCCAAAGAACTCTTCCAAGAGATGGTCTCTCAAGGTGTTCATCCTAATATTGTAACCTACAAAATTTTGATGGATGGTCGATGAAGCTTGGGATCTA 1095
RFL6              GTTGCCAAAGAACTCTTCCAGGAGATGGTCTTTGTTGGTGTGCCACCCAGTGTTGTGACTTATAGTATTTTGCTGGATGGTCGATGATGCTTGGGATCTA 1097
ClustalConsensus  ** ***************** ********** *   ***** *  ** * * **** ** ** *  ****** ************** ************ 952

                        1110      1120      1130      1140      1150      1160      1170      1180      1190      1200
                  ....|....|....|....|....|....|....|....|....|....|....|....|....|....|....|....|....|....|....|....|
RFL11             TTTTGTAGCCTCCCTCTCAAAGAAGTGAAGCCTGATGTCAAAACATACACCATAATGATTGGAGGACTATGTAAGAAAGGCTCGATGTCTAAAGCGGGCC 1195
RFL6              TTCTGTAGCCTCCCTCTCAAAGGAGTGAAGCCTGAAGTCAGAACATATAATATAATGATTGGAGGATTATGTAAGAAAGGCTCGCTGCCTGAAGCGGTCT 1197
ClustalConsensus  ** ******************* ************ **** ****** *  *************** ***************** ** ** ****** *  1039

                        1210      1220      1230      1240      1250      1260      1270      1280      1290      1300
                  ....|....|....|....|....|....|....|....|....|....|....|....|....|....|....|....|....|....|....|....|
RFL11             TGTTATTTAAGAAAATGGGAGAGGATGGGATTGCACCAGATGATTGTACATGGAACACACTAATCCGAGCTCATCTCCGAGGTGGTGACTTAGCAACATC 1295
RFL6              TGTTGTTTAGAAAGATGGGAGAGGCTGGGGTTGCGCCAAGCAGTGGTACATACAACACACTAATACGAGCTCATCTCAGAGGTGGTGACTTAACAAAATC 1297
ClustalConsensus  **** ****  ** ********** **** **** ***     * ******  *********** ************ ************** *** *** 1120

                        1310      1320      1330      1340      1350      1360      1370      1380      1390      1400
                  ....|....|....|....|....|....|....|....|....|....|....|....|....|....|....|....|....|....|....|....|
RFL11             AGCTAAACTTATAGAAGAAATGAAGAGTTGTGGGTTCTCTGCAGATGCTTCACATAAAGATTGTTATGGATATGTTATGGATGGTAGAATGAAGAAAAGC 1395
RFL6              AGCTGAACTTATCGAAGAAATGAAGAGGTGTGGGTTCTCTGCAGATGCTTCACATAAAGATTGTTATGGATATGTTATGGATGGTAGAATGAAGAAAAGC 1397
ClustalConsensus  **** ******* ************** ************************************************************************ 1217

                        1410      1420
                  ....|....|....|....|
RFL11             TTTCTGGATTGCTTTCTTAG 1415
RFL6              TTTCTGGATTGCTTTCTTAG 1417
ClustalConsensus  ******************** 1237
```

Figure S6. Sequence alignment of *BnaRFL11* with *BnaRFL6*.
