# Supplementary material for: Induction of Male Sterility by Targeted Mutation of a Restorer-of-Fertility Gene with CRISPR/Cas9-Mediated Genome Editing in Brassica napus L
Source: Plants (Basel). 2022 Dec 13;11(24):3501. doi: 10.3390/plants11243501 (PMC9785856; doi:10.3390/plants11243501)
Supplement: Supplementary file 1 [file plants-11-03501-s001.zip › Table S1.pdf]

Primer name	Primer sequence (5′→3′)
**The primer used in two-sgRNA vector construction**	
cpa-1sf	ATATATGGTCTCGATTGCAAGACTCAGAGGGATACGGTT
cpa-1f0	TGCAAGACTCAGAGGGATACGGTTTTAGAGCTAGAAATAGC
cpa-2r0	AACCCAAGACTCAGAGGGATACCAATCTCTTAGTCGACTCTAC
cpa-2sr	ATTATTGGTCTCGAAACCCAAGACTCAGAGGGATACCAA
**The primer used for colony PCR and sequencing**	
U626-IDF	TGTCCCAGGATTAGAATGATTAGGC
U629-IDR	AGCCCTCTTCTTTCGATCCATCAAC
**Primers used in the present study for positive transgenic detection.**	
CPA TOP	TCACTGAAGATGAAGATGGGAGTGGAG
CPA BOTIM	CGACGATCCCACTTCGCAATCTC
Cas9-F888	ATGGCTCCGAAGAAGAAGAGGAAG
Cas9-R888	GGCCAGGAGGTTATCCAGGTCA
**Primers used in the present study for transgenic mutation detection**	
CPA TOP	TCACTGAAGATGAAGATGGGAGTGGAG
CPA BOTIM	CGACGATCCCACTTCGCAATCTC
**Primers used in the present study for transgenic mutation detection based on SNP**	
13550 F CMT	CTTACACTAGACGCTGAGAGAGATCTTGAT
13550 R CMT	CCCACACGTCTTCACCACTCAAG
**Primers used in the present study for off-target Detection in BnaPPR**	
ZUN21-F	GGAGTGAGTACGGTGTGCGAAATAACTATCATTTTCTTACG
ZUN21-R	GAGTTGGATGCTGGATGGCGCGAGTCAAACCGGATTC
ZUN22-F	GGAGTGAGTACGGTGTGCAAACAATTATCATTTTCTTACG
ZUN22-R	GAGTTGGATGCTGGATGGGGTTTCAATTACGCGAGTC
ZUN23-F	GGAGTGAGTACGGTGTGCCAATCCATACTTTTCCTCTTGG
ZUN23-R	GAGTTGGATGCTGGATGGCGAGAGAGACACAAGGTAG
ZUN24-F	GGAGTGAGTACGGTGTGCCACTACATATGCATATTCTAC
ZUN24-R	GAGTTGGATGCTGGATGGCATAAGTGAGAACAACCTTTG
ZUN25-F	GGAGTGAGTACGGTGTGCAAGAGTTCCCAACTATCATAT
ZUN25-R	GAGTTGGATGCTGGATGGAATATACGTTGTATCATACAA
**Primers used in the present study positive detection of Overexpression plants**	
35sf left	CATTTCATTTGGAGAGGACGTCGA
35 cpaRig.OE	CTCCACAGCTTCAGAGACTCGAC
**Primers used in the present study for qRT-PCR**	
cpaL.OE	GATTGGTACGTGAGCCTCGTATCC
cpaR.OE	CGTGTAGAGGTCATGCGCAATC
**The primer used in cytoplasm identification**	
MSS2-L	CTGAAGTTGAAAAAGGGAATGT
MSS2-R	TTGATCAAGACGAGGAGTAGCT

Primer name	Primer sequence (5′→3′)
MSS4-L	AGTATTTCGTTCACCTTGGC
MSS4-R	GCTTGGTGGAAAAGATCGTA
MSS13-L	TAGCGCTATCTTTCGGCCCT
MSS13-R	ACCCGAAGGTCCTGGTCTCT
MSS14-L	GCTCGTTCGATTAAGCTCAA
MSS14-R	GAATTCCTCTTTCATTGCGG
**The primer used in nap-CMS qRT-PCR**	
nad5	GACGATCGATTATCTACCCA
	CTATCATTATGCCTTTGCAA
atp6	ACCAAGGATTGCCAAGCATT
	ATCCATCTTTGTTCATGCTGC
Orf222	ATGATGGTGACCACATCTGCT
	GCAATCGTCCCTACCGTCGTA
Orf139	ATGAAAGAATCATTTTATGCT
	TATCGAAGTCCTCTCCTTCA
Ap3	TACCACCATCTGAAATGCGCG
	CATTTGCTGAGATACTGGATG

Table S1. The primer used in all studies.
