# Supplementary material for: Induction of Male Sterility by Targeted Mutation of a Restorer-of-Fertility Gene with CRISPR/Cas9-Mediated Genome Editing in Brassica napus L
Source: Plants (Basel). 2022 Dec 13;11(24):3501. doi: 10.3390/plants11243501 (PMC9785856; doi:10.3390/plants11243501)
Supplement: Supplementary file 1 [file plants-11-03501-s001.zip › Table S2.pdf]

**Table S2. Genotypical study of *bnarfl11* transgenic plants and next-generation transmission analysis.**

| | | | *BnaRFL11* | |
|---|---|---|---|---|
| **Plant ID #** | **Progeny** | **+ve** | **S1** | **S2** |
| ***bnarfl11*-1** | $T_0$ | Yes | Homo (+2bp) | Homo (+1bp, -1bp) |
| ***bnarfl11-1*-5** | $T_1$ | Yes | Homo (+2bp) | Homo (+1bp, -1bp) |
| ***bnarfl11*-5** | $T_0$ | Yes | Homo (-31bp) | Homo (-5bp, +11bp) |
| ***bnarfl11*-5-8** | $T_1$ | Yes | Homo (-31bp) | Homo (-5bp, +11bp)) |
| ***bnarfl11*-8** | $T_0$ | Yes | Homo (-3bp) | Hetero |
| ***bnarfl11*-8-3** | $T_1$ | Yes | Homo (-3bp) | Hetero |
| ***bnarfl11*-15** | $T_0$ | Yes | Hetero | Homo (-9bp) |
| ***bnarfl11*-15-3** | $T_1$ | Yes | Hetero | Homo (-9bp) |
| ***bnarfl11*-19** | $T_0$ | Yes | Homo (-143bp) | Homo (-11bp) |
| ***bnarfl11*-19-8** | $T_1$ | Yes | Homo (-143bp) | Homo (-11bp) |
| ***bnarfl11*-23** | $T_0$ | Yes | Homo (+2bp) | Homo (-18bp) |
| ***bnarfl11*-23-4** | $T_1$ | Yes | Homo (+2bp) | Homo (-18bp) |
| ***bnarfl11*-27** | $T_0$ | Yes | Homo (+2bp) | Homo (-2bp) |
| ***bnarfl11*-27-4** | $T_1$ | Yes | Homo (+2bp) | Homo (-2bp) |
| ***bnarfl11*-34** | $T_0$ | Yes | Homo (-2bp) | Hetero |
| ***bnarfl11*-34-8** | $T_1$ | Yes | Homo (-2bp) | Hetero |
| ***bnarfl11*-38** | $T_0$ | Yes | Homo (-4bp) | Homo (+1bp, -2bp) |
| ***bnarfl11*-38-10** | $T_1$ | Yes | Homo (-4bp) | Homo (+1bp, -2bp) |
| ***bnarfl11*-44** | $T_0$ | Yes | Homo (+2bp) | Homo (+2bp) |
| ***bnarfl11*-44-7** | $T_1$ | Yes | Homo (+2bp) | Homo (+2bp) |
| ***bnarfl11*-57** | $T_0$ | Yes | Homo (+3bp) | Homo (+2bp, -4bp) |
| ***bnarfl11*-57-3** | $T_1$ | Yes | Homo (+3bp) | Homo (+2bp, -4bp) |
| ***bnarfl11*-69** | $T_0$ | Yes | Homo (+6bp, -2bp) | Homo (-5bp) |
| ***bnarfl11*-69-5** | $T_1$ | Yes | Homo (+6bp, -2bp) | Homo (-5bp) |
| ***bnarfl11*-73** | $T_0$ | Yes | Hetero | Homo (-8bp) |
| ***bnarfl11*-73-5** | $T_1$ | Yes | Hetero | Homo (-8bp) |
| ***bnarfl11*-75** | $T_0$ | Yes | Homo (-6bp, +3bp) | Homo (+1bp, -2bp) |
| ***bnarfl11*-75-6** | $T_1$ | Yes | Homo (-6bp, +3bp) | Homo (+1bp, -2bp) |
| ***bnarfl11*-79** | $T_0$ | Yes | Homo (-8bp, +5bp) | Hetero |
| ***bnarfl11*-79-7** | $T_1$ | Yes | Homo (-8bp, +5bp) | Hetero |
| ***bnarfl11*-83** | $T_0$ | Yes | Hetero | Homo (+4bp, -1bp) |
| ***bnarfl11*-83-4** | $T_1$ | Yes | Hetero | Homo (+4bp, -1bp) |
| ***bnarfl11*-87** | $T_0$ | Yes | Homo (+2bp) | Homo (-4bp) |
| ***bnarfl11*-87-7** | $T_1$ | Yes | Homo (+2bp) | Homo (-4bp) |

Hetero means heterozygous, Homo means homozygous, and WT means wild form. "-/+" demonstrates the synchronized removal and addition, in each copy of the mutated representative target, of the indicated number of nucleotides; All other targets are wild type except indicated targets.
