# Supplementary material for: Induction of Male Sterility by Targeted Mutation of a Restorer-of-Fertility Gene with CRISPR/Cas9-Mediated Genome Editing in Brassica napus L
Source: Plants (Basel). 2022 Dec 13;11(24):3501. doi: 10.3390/plants11243501 (PMC9785856; doi:10.3390/plants11243501)
Supplement: Supplementary file 1 [file plants-11-03501-s001.zip › Table S3.pdf]

Table S3. Molecular investigation of CRISPR/Cas9-tempted transmutations in *bnarfl11* and it has spread to from T_0 to T_1 generations.

Examination of T_0 lines			Mandilion Segregation of mutation to T_1 lines				
**Plant identification**	**Genetic constitution**	**Mutation [a]**	**plants examined**	**WT**	**Hetero**	**Homo**	**transmission rate[b]**
***bnarfl11*-1**	AA09	+2, +1, -1	14	AA (0)	Aa (0)	aa (14)	100%
***bnarfl11*-5**	AA09	+1, -31, -1, +1	14	AA (0)	Aa (0)	aa (14)	100%
***bnarfl11*-8**	AA09	-3	14	AA (0)	Aa (7)	aa (7)	50%
***bnarfl11*-15**	AA09	-9, +1	14	AA (0)	Aa (5)	aa (0)	35%
***bnarfl11*-19**	AA09	-143, -11	14	AA (0)	Aa (0)	aa (14)	100%
***bnarfl11*-23**	AA09	+2, -18	14	AA (0)	Aa (0)	aa (14)	100%
***bnarfl11*-27**	AA09	+2, -2	14	AA (0)	Aa (0)	aa (14)	100%
***bnarfl11*-34**	AA09	-22, +1	14	AA (0)	Aa (7)	aa (7)	50%
***bnarfl11*-38**	AA09	-4, +1, -2	14	AA (0)	Aa (0)	aa (14)	100%
***bnarfl11*-44**	AA09	+2, -7, +2	14	AA (0)	Aa (0)	aa (14)	100%
***bnarfl11*-57**	AA09	+1, -1	14	AA (0)	Aa (0)	aa (14)	100%
***bnarfl11*-69**	AA09	+1, -2, -5	14	AA (0)	Aa (0)	aa (14)	100%
***bnarfl11*-73**	AA09	0	14	AA (7)	Aa (7)	aa (0)	50%
***bnarfl11*-75**	AA09	-6, +3, +1, -2	14	AA (0)	Aa (0)	aa (14)	100%
***bnarfl11*-79**	AA09	-8, +5	14	AA (0)	Aa (7)	aa (7)	50%
***bnarfl11*-83**	AA09	+4, -1, -2	14	AA (0)	Aa (7)	aa (7)	50%
***bnarfl11*-87**	AA09	+2, -11, -4, +2	14	AA (0)	Aa (0)	aa (14)	100%

* WT stands for wild type; Hetero stands for heterozygous; Homo stands for homozygous, a "- and +" denotes the loss and addition of the specified number bases simultaneously. B was determined based on the number of lines with mutation by the entire line verified for mutation.
