# Supplementary material for: Induction of Male Sterility by Targeted Mutation of a Restorer-of-Fertility Gene with CRISPR/Cas9-Mediated Genome Editing in Brassica napus L
Source: Plants (Basel). 2022 Dec 13;11(24):3501. doi: 10.3390/plants11243501 (PMC9785856; doi:10.3390/plants11243501)
Supplement: Supplementary file 1 [file plants-11-03501-s001.zip › Table S4.pdf]

Table S4. Exposure of off-effects for designed guiding-RNA in mutant lines.

Sequence ID		Sequence	No. of mismatching bases	assumed off-target locus	Plant number sequenced	Off-target deletion (Y/N)
**On target *BnaRFL11***	**S1**	**CAAGACTCAGAGGGATACGAGG**				
Off-target site *BnaRFL11*	OFF-1	GAAGAATTAGAGGGATACG	3	*BnaA05g25250D*	50	No
	OFF-2	GAAGACAAAGAGGGATACG	3	*BnaC06g22890D*	50	No
	OFF-2	GAAGACAAAGAGGGATACG	3	*BnaA01g20240D*	50	No
	OFF-4	CTTGAATCATAGGGATACG	4	*BnaC03g01350D*	50	No
**On target *BnaRFL11***	**S2**	**GTATCCCTCTGAGTCTTGGAGG**			50	No
Off-target site *BnaRFL11*	OFF-1	TTATTCGTCTGAGTCTTGG	3	*BnaC09g23000D*	50	No
	OFF-2	GAGTCACGCTGAGTCTTGG	4	*BnaCnng39820D*	50	No
	OFF-3	GAGTCACGCTGAGTCTTGG	4	*BnaC09g14400D*	50	No
