# Supplementary material for: Induction of Male Sterility by Targeted Mutation of a Restorer-of-Fertility Gene with CRISPR/Cas9-Mediated Genome Editing in Brassica napus L
Source: Plants (Basel). 2022 Dec 13;11(24):3501. doi: 10.3390/plants11243501 (PMC9785856; doi:10.3390/plants11243501)
Supplement: Supplementary file 1 [file plants-11-03501-s001.zip › Table S5.pdf]

Table S5. Comparison of editing efficiency for *BnaRFL11*-promoters *with* other published gene-editing studies.

Promoter of sgRNA	Target sequence	Target genes[b]	GC % content (Without PAM)	Positive lines of T_0	Editing lines	Editing efficiency[c]
U6-26	**CAAGACTCAGAGGGATACGAGG**	***BnaRFL11***	**57%**	**108**	**70**	**64.81%**
	GAGGTCGTCCGAGATGGCCGAGG	*BnaA9-RGA*	70%	29	28	96.60%
	CAAGGTGAGGTCGTCAGAGATGG	*BnaC9-RGA*	55%	29	25	86.20%
	CAAGGTTAGGTCTTCGGAGATGG	*BnaA6-RGA*	50%	29	28	96.60%
	CAGGTCGTCGGAGATGGCTGAGG	*BnaC7-RGA*	65%	29	27	93.10%
	GCACAAGACGACGGCATTGCTGG	*BnaA2-DA2*	60%	17	7	41.20%
	GAACACCTTAGGATTTGTGCTGG	*BnaC5-RGA*	50%	17	6	35.30%
U6-29	**GTATCCCTCTGAGTCTTGGAGG**	***BnaRFL11***	**50%**	**108**	**50**	**46.29%**
	ACCCGTCGGAGCTCTACTCGTGG	*BnaA9-RGA*	65%	29	8	27.6%
	ACCCGTCGGAGCTTTACTCGTGG	*BnaC9-RGA*	60%	29	9	31.00%
	ACCCCGCTGAGCTTTACTCGTGG	*BnaA6-RGA*	60%	29	10	34.50%
	ACCCTGCTGAGCTCTACTCTTGG	*BnaC7-RGA*	55%	29	12	41.40%
	GGGAGCTAAGTACGTAGACTTGG	*BnaA2-DA2*	50%	17	8	47.12%
	GAACACCTTAGGATTTGTGCTGG	*BnaC5-RGA*	45%	17	14	82.40%

[a]The PAM is in red.. [b]The target genes *BnaRFL11* (Current study), *BnaA9, BnaC9, BnaA6, BnaC7, BnaA2, BnaC5* (Yang et al 2018). [c] for each target, the %age of modified plantations compared to the number of tested plants.
